# Inter-laboratory comparison of a serum fibroblast growth factor receptor 3 (FGFR3) antibody test in sensory neuropathies

**DOI:** 10.3389/fimmu.2025.1604456

**Published:** 2025-10-10

**Authors:** Luise Appeltshauser, Christian P. Moritz, Lena Reinhardt, Luisa Kreß, Nurcan Üçeyler, François Lassablière, Anastasia Barcic, Sabine Seefried, Claudia Sommer, Yannick Tholance, Jean-Christophe Antoine, Jean-Philippe Camdessanché, Kathrin Doppler

**Affiliations:** ^1^ Department of Neurology, University Hospital Würzburg, Würzburg, Germany; ^2^ Department of Neurology, CHU Saint-Etienne, Saint-Etienne, France; ^3^ Synaptopathies et autoanticorps (SynatAc), Institut MELIS-NeuroMyoGène, INSERM U1314/CNRS UMR 5284, Université Jean Monnet, Saint-Étienne, France; ^4^ Department of Biochemistry, CHU Saint-Etienne, Saint-Etienne, France

**Keywords:** fibroblast growth factor receptor 3, ELISA, autoantibodies, sensory neuropathy, sensory neuronopathy, small fiber neuropathy, dorsal root ganglion

## Abstract

**Introduction:**

Autoantibodies against fibroblast growth factor receptor 3 (FGFR3) have been suggested as a diagnostic marker in both sensory large and small fiber neuropathy. Yet, their clinical relevance remains unclear and no standardized protocols for antibody testing exist. Here, we evaluate an anti-FGFR3 ELISA protocol in an inter-laboratory comparison.

**Methods:**

We performed anti-FGFR3 ELISA on 42 serum samples of patients with sensory neuronopathy (n = 18), small fiber neuropathy (n = 18), and healthy controls (n = 6) in two independent centers in France (center 1) and Germany (center 2) using identical protocols, with double immunofluorescence staining on rat dorsal root ganglion (DRG) sections as a confirmational test.

**Results:**

Overall ELISA concordance was 34/42 (81.0%, Cohen’s kappa = 0.61, substantial agreement). Discordance occurred for sera with optical densities (OD) near the cut-off. ODs correlated (r = 0.68, p < 0.0001), but were lower at center 2 (median = 0.076 vs 0.293, p < 0.0001), indicating that cut-off values are laboratory-specific. 11/16 (68.8%) ELISA-double-positive sera stained small DRG neurons, colocalizing with commercial anti-FGFR3 antibody, while positive binding was only found in 1/20 (5%) of ELISA-negative sera (p < 0.0001). DRG-positive samples showed higher ODs than negative ones (p < 0.0001).

**Discussion:**

We provide and evaluate a detailed ELISA protocol for anti-FGFR3 diagnostic assessment. Positive results near the threshold should be interpreted cautiously. Anti-FGFR3 DRG staining may be a useful confirmatory method and could increase diagnostic specificity. This study facilitates future studies on the diagnostic relevance of anti-FGFR3 autoantibodies in sensory neuropathies.

## 1 Introduction

Sensory neuropathies, encompassing both large and small fiber pathology, are debilitating diseases of the peripheral nervous system (PNS), characterized by sensory deficits, autonomic dysfunction, and/or neuropathic pain (1, 2). The etiologies are diverse, including metabolic, paraneoplastic, genetic, toxic, and autoimmune causes. Despite extensive diagnostic work-up, many cases remain idiopathic (1, 3). Identifying autoimmune etiologies among these idiopathic cases is particularly crucial, as this could have a direct impact on treatment decisions (2). Recent studies have identified autoantibodies against fibroblast growth factor receptor 3 (FGFR3) as a potential biomarker for sensory neuropathies particularly affecting the dorsal root ganglia (DRG), such as sensory neuronopathy (SNN) and small fiber neuropathy (SFN) (3–5).

FGFR3, a member of the tyrosine kinase receptor family, is suggested to be expressed in sensory neurons of the DRG and plays an important role in neuronal development and maintenance (4, 6, 7). The presence of anti-FGFR3 antibodies in SNN and SFN could indicate a possible autoimmune mechanism targeting the DRG. Still, the antibody’s ability to bind these targets and their clinical relevance remain unclear. Further, anti-FGFR3 are not exclusive for sensory neuropathies, but can also occur in other neuropathies, questioning the specificity of the test (8).

Detection of anti-FGFR3 is primarily conducted using ELISA, and normalization of serum-specific background noise by subtraction can increase the sensitivity and specificity of the assay (4, 9). Still, many studies report anti-FGFR3 test results without a published protocol or a second validation method. These inconsistencies underscore the necessity for standardized testing protocols to ensure reliable and reproducible results.

In this context, we aimed to evaluate an anti-FGFR3 ELISA protocol through an inter-laboratory comparison between two independent centers in France and Germany. By analyzing serum samples from patients with SNN, SFN, and healthy controls, we sought to assess the concordance of ELISA results and establish standardized procedures for antibody testing. Additionally, we employed double immunofluorescence staining on DRG sections, providing a complementary method to enhance diagnostic accuracy. Our findings address the critical need for standardizing anti-FGFR3 testing in sensory neuropathies.

## 2 Methods

### 2.1 Anti-FGFR3 ELISA protocol

We established and applied a protocol for FGFR3 testing from the reference laboratory (center 1) at center 2 according to previously published information only (4), and in a second step adapted the protocol to the details provided by center 1, as shown in **Table 1**. For center 1, cut-off values to define an anti-FGFR3-positive result had been determined as previously described from 65 healthy control sera without reported autoimmune disease, neurological disease including neuropathic pain, or malignancy (4). For center 2, these cut-off values were determined using 50 healthy controls whose sera had been recruited during previous studies (10, 11) or routine diagnostic testing.

**Table 1:** Standardized ELISA protocol for anti-FGFR3 testing.

Category	Reagent	Composition and dilution
*ELISA plate*	Maxisorb 96-well plates	#10394751, Thermo Fisher Scientific, Waltham, Massachusetts, USA
*solutions and buffer*	blocking solution	0.1% fish gelatine (#G7041-100G Merck, St. Louis, MO) 0.06% Tween-20 (#P1379-250ML, Merck), 3% BSA (#A4503-50G, Merck) in PBS (#D1408, Merck)
	washing solution	0.1% Tween20 in PBS
	coating buffer	0.05 M carbonate bicarbonate (#C3041, Merck)
	OPD substrate	0.4 mg/ml o-Phenylendiamin –dihydrochloride (OPD, # P6662, Merck) in 0.05 M citrate-phosphate buffer (#P4809, Merck) with 0.012% H_2_O_2_
*protein and antibodies*	FGFR3 protein	1 µg/ml in coating buffer (#PR4631B or #PV4107, Thermo Fisher Scientific, Waltham, MA)
	rabbit anti-FGFR3	1:3000 (0.33 µg/ml) in blocking solution (#GTX31549, Genetex, Irvine, CA)
	human serum samples	1:50 in blocking solution
	Polyclonal Swine Anti-Rabbit IgG/HRP	1:1000 in blocking solution (#P217, Agilent, Santa Clara, CA)
	Polyclonal Rabbit Anti-Human IgG/HRP	1:5000 in blocking solution (#P0214, Agilent)
Category	Protocol	
*plate layout (per plate)*	- all human samples: perform serum-specific background normalization (two wells with protein coating and one well with coating buffer only, not next to each other) - include desired number of human test samples - include one positive human sample (if available) and one negative control sample (healthy control serum) - include one background control (blocking solution only) and one commercial positive control (rabbit anti-FGFR3, see above), in duplet assessment each	
*coating*	- 100 µl protein/coating buffer per well - incubation at 4°C overnight	
*washing and blocking*	- two times with 300 µl washing buffer per well, alternate directions - add 300 µl of blocking solution and incubate for 2 h at RT and constant agitation - wash four times with 300 µl washing buffer per well, alternate directions	
*serum/primary antibody incubation*	- 100 µl of test sample/background control/negative control/positive control per well - incubation at 4°C overnight - wash six times with 300 µl washing buffer per well	
*secondary antibody incubation*	- 100 µl of corresponding secondary antibody (anti-rabbit/anti-human) per well - incubation at 4°C for 2 h - wash ten times with 300 µl washing buffer per well, alternate directions	
*substrate reaction and readout*	- add 100 µl of OPD substrate - incubation for 30 minutes at 37°C in the dark - read in ELISA reader at 440 nm	
*evaluation*	- use plate only if negative/positive controls worked - calculate the mean value for each sample - perform serum-specific background noise normalization (subtract serum-specific value with coating buffer only from mean protein-coated value) - measure at least n = 30 healthy control sera - positive results: > 3 SD above the mean of controls - confirm positive results with two consecutive ELISAs - final result: mean value of all ELISAs (if consistent results) - final definition of anti-FGFR3-seropositivity: three consecutive positive results	

We provide a detailed and structured version of a recent ELISA protocol for anti-FGFR3 testing (4), including substances and origin and evaluation methods using serum-specific background normalization for analysis (9).

### 2.2 Patients

**Figure 1:**
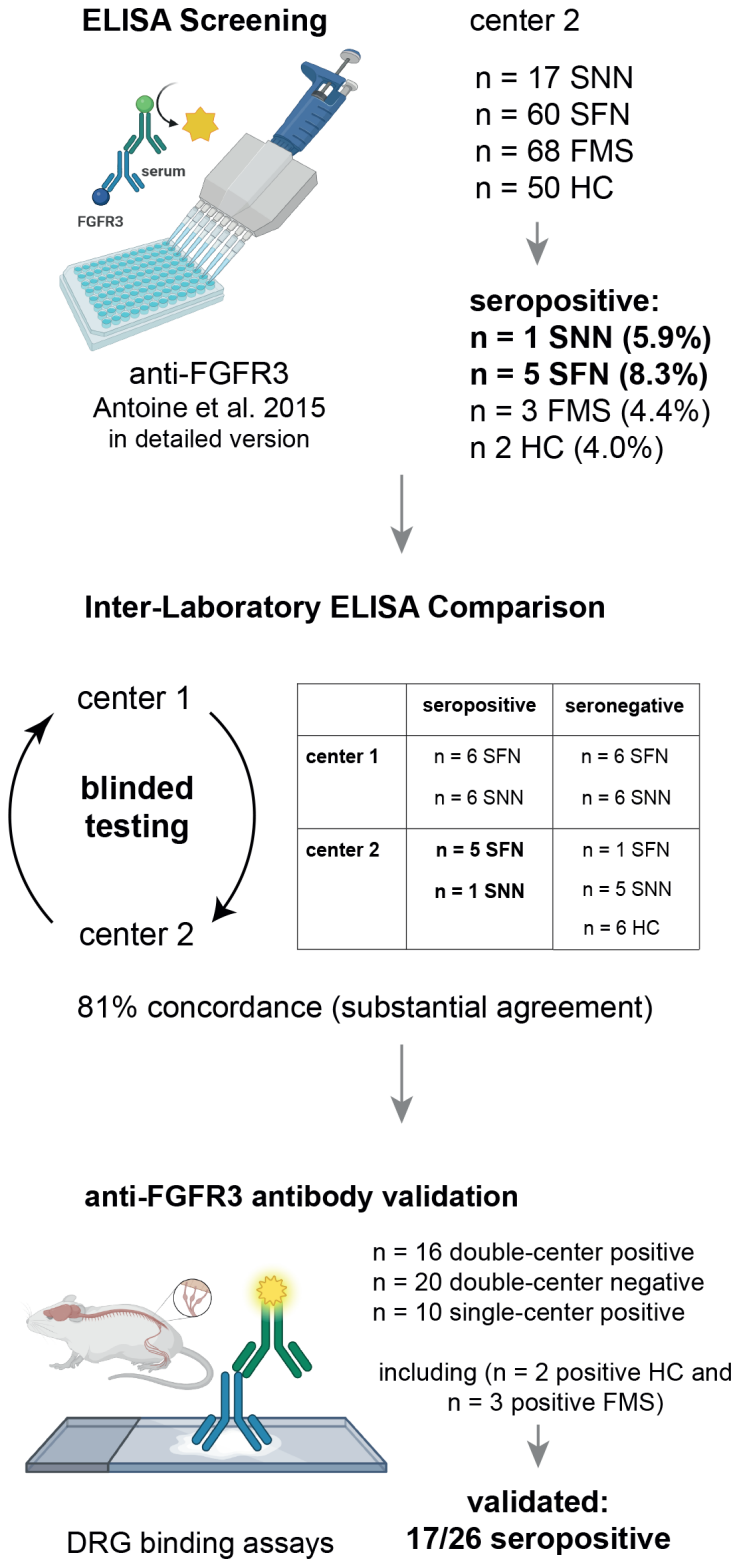
Schematic illustration of the study design. DRG, Dorsal Root Ganglion; FGFR3, Fibroblast Growth Factor Receptor 3; FMS, Fibromyalgia Syndrome; HC, healthy controls; pat., patients; SFN, Small Fiber Neuropathy; SNN, Sensory Neuronopathy.

A schematic illustration of the study design including patient and control cohort sizes is shown in **Figure 1**. To screen for anti-FGFR3 antibodies at center 2, we included sera of 60 patients with SFN fulfilling the diagnostic criteria of “definite SFN” proposed bei Devigili et al. (12, 13), namely clinical symptoms of small fiber damage, exclusion of large fiber pathology by sural nerve conduction studies, and either abnormal results in quantitative sensory testing, or reduced intraepidermal nerve fiber density in skin biopsy at the ankle, or both. Further, we included sera of 17 patients with SNN who fulfilled the diagnostic criteria proposed by Camdessanché et al. (14) (n = 11 possible, n = 5 probable). As a control group, we assessed 68 patients with fibromyalgia syndrome (FMS) diagnosed according to the American College of Rheumatology (ACR) 1990 or ACR 2010 classification without further autoimmune disease, polyneuropathy, or pain of other origin as previously reported (15). Of these FMS patients, intraepidermal nerve fiber density was reduced in 33/68 (proximal) or 30/68 (distal) individuals (15).

For the inter-laboratory comparison study, we included all seropositive patient samples from center 2 detected by the screening assay (n = 6), and matched them with equal numbers of seronegative sera, healthy controls (n = 6), and samples from center 1 recruited in previous studies (4, 9). This resulted in a cohort of 36 patients with sensory neuropathies (18 with SFN and 18 with SNN), each fulfilling the respective diagnostic criteria (12, 14), of whom 18 had been considered seropositive and 18 had been considered seronegative. Demographic data of patients included in the inter-laboratory testing are shown in **Table 2**. The study was approved by the Ethics Committees of the Universities of Würzburg and St.-Étienne (reference number 220/20 and IRBN742021/CHUSTE, respectively). Patients and controls either gave informed consent or in case they were lost to follow-up, the Ethics Committee of the University of Würzburg or St.-Étienne approved the use of diagnostic serum samples and retrospective analysis of documented clinical data (reference 2021031501).

**Table 2:** Cohort and demographic data of patients included in the inter-laboratory validation study for an ELISA FGFR3 antibody test.

Center	Seropositive	Seronegative	Mean age (range)	Sex (female/male)
center 1 (France)	n = 6 SFN n = 6 SNN	n = 6 SFN n = 6 SNN	58.2 (36 – 84) 55.2 (35 – 75)	6/6 6/6
center 2 (Germany)	n = 5 SFN n = 1 SNN n = 0	n = 1 SFN n = 5 SNN n = 6 HC	48.6 (42 – 54) 55.6 (45 – 74) 54.8 (51 – 62)	4/2 4/2 4/2
total	n = 11 SFN n = 7 SSN n = 0	n = 7 SFN n = 11 SSN n = 6 HC	55.0 (36 – 84) 55.3 (35 – 75) 54.8 (51 – 62)	10/8 10/8 4/2

HC, healthy control; SFN, Small Fiber Neuropathy; SNN, Sensory Neuronopathy.

### 2.3 Inter-laboratory comparison of ELISA

Patient and control sera were sent to the other center on dry ice and stored at -20°C or -80°C until testing. ELISA using the protocol described in **Table 1** was performed three times in independent assays with serum samples from the other center, and evaluated by blinded researchers. Sera which showed three consecutive values above 3 standard deviations (SD) of the means of controls were considered positive. The degree of positivity was assessed semi-quantitatively according to the mean optical density (OD) level (weakly positive if 3–5 SD and strongly positive if > 5 SD above the mean of controls).

### 2.4 Immunofluorescence staining

To confirm ELISA results for anti-FGFR3 antibodies, 41/42 sera included in the inter-laboratory validation study (n = 1 one serum missing due to insufficient volumes) as well as sera tested positive at center 2 during screening (n = 3 FMS and n = 2 healthy control, see **Figure 1**) were stained on 5-µm cryosections of rat DRG in center 2. After fixation for 10 minutes in acetone at -20°C and blocking with 4% normal goat serum, 4% fetal calf serum, and 0.3% Triton-X-100 in Phosphate-Buffered Saline for one hour at room temperature (RT), serum diluted 1:500 in blocking solution, rabbit Anti-FGFR3 (1:1000, # GTX31549, Genetex, Irvine, CA) and Alexa-647-labeled mouse anti-peripherin (1:1000, # sc-377093, Santa Cruz Biotechnology, Dallas, TX) were incubated overnight at 4°C. After washing, Cy3-labeled anti-human and Alexa-488-labeled anti-rabbit IgG secondary antibodies (1:300, Jackson Immuno Research, Westgrove, PA) were incubated for 1h at RT. Slides were mounted with Vecatshield Mounting Medium with DAPI 1.5 µg/ml (Vector Laboratories, Newark, CA) and assessed using a fluorescence microscope (Zeiss Axiovert 200m, Oberkochen, Germany). Cross-reaction was ruled out by performing single stainings with the respective serum/commercial antibody and by using healthy sera as controls. Staining clusters and colocalization of serum reactivity with commercial anti-FGFR3 and peripherin reactivity were assessed from photomicrographs semiquantitatively by two independent and blinded researchers. For display, identical image modification protocols were applied to photomicrographs of both patient and control sera using *ImageJ* (16). Further, we aimed to establish cell-based assay (CBA) using plasmids for full-length human FGFR3 and the extracellular domain of FGFR3 as previously described (4, 11), including double-staining with patient sera and commercial Anti-FGFR3 (Genetex), but results were not reliably reproducible for positive sera. Therefore, CBA was not applied as further confirmation assay in this study.

### 2.5 Statistics

The frequency of positive/negative results between testing via a self-established protocol and the detailed protocol (**Table 1**) was assessed using Fisher’s exact test. Inter-laboratory concordance of positive/negative results was calculated using Cohen’s Kappa coefficient. OD values between centers were compared using the Mann-Whitney test and Spearman correlation, or t-test depending on normal distribution. DRG binding rates were compared by Chi-square test, and ELISA sensitivity and specificity was calculated by Fisher’s exact test. Testing and display were performed using Prism V9.3.0 (GraphPad Software, San Diego, CA).

## 3 Results

### 3.1 Anti-FGFR3 testing results depend on ELISA protocol

First, we established an anti-FGFR3 ELISA at center 2 using previously published information only (4). The ELISA cut-off value from n = 50 healthy controls for a positive result was at an OD_440_ of 0.102 (0.020 + 3x standard deviation of 0.027). Using this self-established protocol, 1/17 (5.9%) patients with SNN and 1/60 (1.7%) patient with SFN were considered seropositive for anti-FGFR3 antibodies, but also 1/50 healthy controls (2%). We then repeated the assay using a very detailed version of the protocol provided by reference center 1, as shown in **Table 1**, and including three reproductions of the ELISA. The cut-off value was at an OD_440_ of 0.122 (0.039 + 3x standard deviation of 0.028). We still identified 1/17 seropositive patients with SNN, but now 5/60 patients with SFN (8.3%, p = 0.08 compared to first protocol), and also 2/50 healthy controls (4%, p = 0.04 compared to first protocol). The two positive controls were re-assessed, and one of them had developed malignancy (pulmonary adenocarcinoma) four years after recruitment, the other was tested positive for anti-cortactin-antibodies (17). Moreover, 3/68 (4.4%) of patients with FMS were tested seropositive (15). These three individuals had normal results in quantitative sensory testing, partially reduced intraepidermal nerve fiber density, but normal results in clinical examination, thus not fulfilling the SFN diagnostic criteria (12, 13). Thus, controls with autoimmune/malignant context and patients with FMS can also show positive FGFR3 ELISA results. Furthermore, this direct comparison of two protocol versions shows that small modifications can impact cut-off values and results.

### 3.2 Inter-laboratory validation of an anti-FGFR3 assay

**Figure 2:**
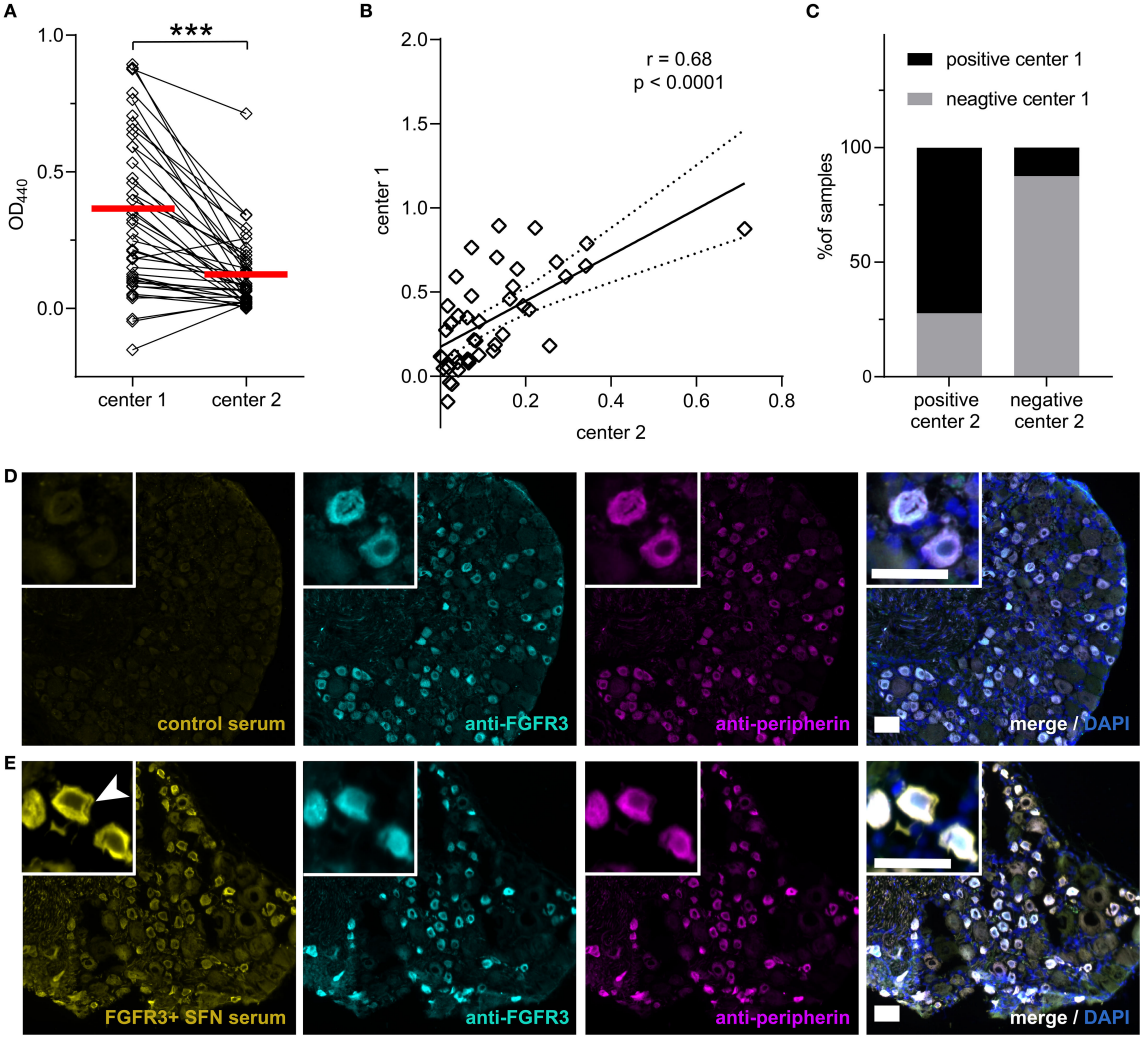
Inter-laboratory anti-FGFR3 ELISA validation and DRG binding. **(A)** Mean OD_440_ for the n = 42 sera included in the inter-laboratory comparison are higher in center 1 than center 2. Respective threshold values are marked in red. **(B)** Mean ODs correlate between the two centers. Regression line is shown as solid and 95% confidence bands as dashed lines. **(C)** Concordance of the overall positive/negative testing results for both centers is displayed in percentage with bar graphs. **(D)** No specific binding of a healthy control serum without FGFR3 antibodies to DRG sections (shown in yellow). Commercial anti-FGFR3 is shown in cyan and anti-peripherin in magenta, without serum colocalization. **(E)** IgG binding of an anti-FGFR3 positive serum of a patient with SFN to DRG sections. Arrowhead marks cytosol of small DRG neurons and membrane specifically bound by patient IgG. Colocalization with commercial anti-FGFR3 and anti-peripherin appears white. Scale bar = 50 µm. DRG, Dorsal Root Ganglion; FGFR3, Fibroblast Growth Factor Receptor 3; OD, optical density; SFN, Small Fiber Neuropathy. ***, p < 0.001.

To validate the anti-FGFR3 ELISA assay, we compared the results of 42 sera between two independent laboratories at center 1 and 2, using identical sera and protocols. Cut-off values for positive results were higher in center 1 compared to those in center 2 (OD_440_ 0.371 vs 0.122), as well as mean ODs in general (median = 0.293 vs 0.076, p < 0.0001, **Figure 2A**). OD values correlated strongly between center 1 and 2 (r = 0.68, p < 0.0001, **Figure 2B**). In total, 34/42 samples (81%) were evaluated in concordance (13 positive, 21 negative, see **Figure 2B**). A Cohen’s kappa coefficient of κ = 0.61 indicated a substantial agreement (18) between both tests. Discordance occurred in one healthy control, four SFN and three SNN sera. Seropositive sera which were evaluated in concordance tended to exhibit higher ODs than sera that were rated as positive only in one center (median of 0.655 vs. 0.537 for those rated positive center 1 only, p = 0.350; median of 0.208 vs. 0.137 for those rated positive in center 2 only, p = 0.08), and 7/8 discordant sera were rated only as weakly positive. In conclusion, we could successfully validate the anti-FGFR3 assay in two independent laboratories with high levels of concordance. Still, cut-off values have to be determined separately for every lab, as mean ODs can vary among different settings.

### 3.3 Anti-FGFR3-positive sera show specific DRG binding patterns

To further validate ELISA results, we performed immunofluorescence binding assays on rat DRG sections with the sera included in the inter-laboratory validation study and FGFR3 seropositive sera from center 2 (including n = 2 positive healthy controls and n = 3 positive FMS sera). Inter-rater comparison revealed almost perfect agreement on semiquantitative assessment and classification of staining cluster regarding the stained cell type (43/46 sera, 93.5%, Cohen’s κ = 0.849). The staining pattern of DRG positive sera included staining of small, peripherin-positive DRG neurons (see **Figure 2E**). Triple immunofluorescence with a commercial anti-FGFR3 antibody revealed colocalized binding of serum, anti-FGFR3, and anti-peripherin on small DRG neurons, but no colocalization in anti-FGFR3 negative sera (see **Figures 2D, E**). While anti-peripherin and commercial anti-FGFR3 stained the cytosol, staining with patient serum also exhibited membrane localization (see **Figure 2E, arrow**).

11/16 (68.8%) ELISA anti-FGFR3-positive sera (confirmed by both centers) and in total 17/26 (65.4%) of single- or double-center positive sera showed binding to DRG small neurons, whereas only 1/20 (5%) sera rated as FGFR3-negative in both centers showed binding to small DRG neurons (p < 0.0001). Out of the 11 anti-FGFR3-positive sera binding small neurons, seven were rated as strongly positive in ELISA in both centers, two as strongly positive in one center (n = 1 center 1 and n = 1 center 2), and two as weakly positive in both centers. Patients with positive DRG neuron staining had higher OD values in the FGFR3 ELISA than patients without specific DRG binding (ELISA at center 1: mean 0.65 vs. 0.23, p < 0.0001; ELISA at center 2: median 0.208 vs. 0.06, p = 0.0011). In general, sera rated as anti-FGFR3-positive in only one center showed higher DRG binding rates than negative sera (3/8, 37.5% vs. 5%, p = 0.026), but a trend towards lower DRG binding-rates compared to double-confirmed positive sera (37.5% vs. 68.8%, p = 0.14). If only double positive (ELISA and DRG staining) were considered as a gold standard for anti-FGFR3 diagnostics, ELISA testing in general (including single-center positive and double-center positive results) showed a sensitivity of 93.3% and a specificity of 61.3%, with a false positive rate of 38.7% (p = 0.0004, Fisher’s exact test). Using DRG staining as a confirmation method, anti-FGFR3 antibodies were not confirmed in FMS sera (0/3 positive), and only in 1/2 healthy control sera tested positive for anti-FGFR3. All other healthy control sera (n = 7) were confirmed negative.

Thus, FGFR3 antibodies can be visualized on small DRG in most patients with high ODs in the anti-FGFR3 ELISA test. The combination of ELISA and binding assays to rat DRG could increase diagnostic specificity for anti-FGFR3 antibodies.

## 4 Discussion

Here, we validate a detailed protocol for an FGFR3 antibody test by ELISA, performed in two independent reference laboratories and introduce test confirmation by tissue-based assays.

This study demonstrates that ELISA is reliable for detecting anti-FGFR3 antibodies. Previous experimental and clinical studies often lacked detailed protocols for determining anti-FGFR3 antibodies, especially when tested in commercial laboratories (8, 19–22). Moreover, commercially available anti-FGFR3 antibody detection kits are costly, are not validated for diagnostic use, and do not include serum-specific background normalization (23, 24). In this study, we did therefore not include those assays for validation. Here, we present comprehensive instructions for a self-established and normalized ELISA, and only consider assays with consistent results from ≥ 3 repetitions as positive. These measures of normalization (9, 25) and repetition enhance both the sensitivity and specificity of the assay. However, our findings indicate that minor variations in the protocol can influence assay accuracy. Prior studies on inter-laboratory assessments of local, non-commercial ELISAs for antibody-mediated peripheral nerve diseases also highlight that different ELISA protocols can affect assay performance (26, 27). Therefore, the detailed protocol presented here could be used as a standard for future antibody testing if further validated in larger studies.

Still, some weakly positive samples yielded discrepant results between the two reference laboratories, indicating that values close to the threshold should be interpreted cautiously. Possible explanations for this include variations in probe handling, experimental conditions (e.g., laboratory temperature), reagent preparation, hardware-specific differences, or variations in human pipetting. Whether these discrepancies represent false positives or false negatives remains unclear, as no independent gold standard is available for anti-FGFR3 diagnostic assessment. To prevent false positive results, future ELISA protocols could include disease controls with an autoimmune context for the calculation of cut-off values and use higher-fold standard deviations for the cut-off due to variability of control values. Consequently, a positive anti-FGFR3 result should always be interpreted in a clinical context, and treatment decisions should not be solely based on antibody test results. Further, independent assays should be developed to increase diagnostic specificity.

Binding to DRG sections can help identify patients with autoantibodies and correlate with clinical symptoms, as demonstrated in previous studies involving cohorts of patients with Guillain-Barré syndrome and FMS (15, 28, 29). FGFR3 is expressed in the cytosol and the nucleus of DRG neurons, and SNN patients with high-titer FGFR3 antibodies bind to the cytoplasm when tested on cultivated rat DRG neurons (4). Here, patients with SFN and SNN strongly positive in ELISA bound to small DRG neurons in cryosections, colocalizing with a commercial anti-FGFR3 antibody, and sera binding to DRG sections showed higher ELISA values than negative sera. Samples that show low OD values in ELISA and cannot be validated by DRG binding assays might lack specificity. Tissue-based immunofluorescence assays are straightforward to perform and serve as a diagnostic standard in autoantibody-mediated neurological diseases (30). Therefore, we recommend using immunofluorescence staining on rat DRG sections as a viable method for further validating anti-FGFR3 results, as it might increase diagnostic specificity.

Although anti-FGFR3 antibodies have been recognized for a decade (4), their specificity, pathogenic role, and impact on treatment decisions in sensory neuropathies remain inconclusive. Initially described in neuropathies predominantly affecting DRG sensory neurons such as SNN and SFN (4, 5), anti-FGFR3 antibodies have now also been detected in chronic inflammatory demyelinating polyradiculoneuropathy, motor neuropathy, trigeminal neuropathy, FMS and patients with corneal neuropathic pain when using commercial anti-FGFR3 testing (8, 15, 21, 31, 32). 48% of the FMS control cohort in this study also showed reduced intraepidermal nerve fiber density as a sign of small fiber pathology, which can occur in up to 50% of patients with FMS (33–35). Still, the FGFR3 seropositive FMS patients did not fulfill diagnostic criteria for SFN, and anti-FGFR3 positive results were not confirmed using tissue-based assays. Further, we detected two control sera with a positive anti-FGFR3 ELISA result, with one of them being confirmed using tissue-based assays. Upon further investigation, these two individuals developed autoimmunity or malignancy after recruitment for this study. These findings indicate that ELISA-based anti-FGFR3 are not specific for sensory neuropathies and could occur in other conditions, but most certainly could also be due to false-positive ELISA results when no confirmation method such as tissue-based assay is applied. Thus, confirmation tests using DRG binding assays could enhance specificity also in other conditions than sensory neuropathies.

Although FGFRs play a crucial role in neuronal development, functioning, and survival (6), and different functional anti-FGFR3 binding epitopes are related to clinical subtypes of SNN (36), no conclusions can be made on a potential pathogenicity of antibodies against FGFR3 in sensory neuropathies. With our current knowledge, anti-FGFR3 antibodies could only be considered a possible marker of an underlying autoimmune context, similar to anti-argonaute antibodies in SNN (37–39). Similarly, the significance of further antibodies in SFN, such as anti-PlexinD1 and anti-trisulfated heparan disaccharide (TS-HDS) (20, 40), is still under investigation, and future studies could benefit from methodological standardization similar to this study before concluding their significance.

Immunomodulatory treatment in patients with anti-FGFR3 and anti-TS-HDS antibodies has not shown any beneficial effect for patients with SFN in a pilot study, with similar results for anti-FGFR3 in retrospective analyses (5, 22, 41). Still, antibody detection methods were not always specified, with a possible impact on the study results.

Here, we provide a standardized and validated protocol for anti-FGFR3 antibody detection, and offer a method for antibody confirmation, thus paving the way for more accurate testing and multicenter investigations to understand the clinical relevance of anti-FGFR3 antibodies in sensory neuropathies.

## Data Availability

The raw data supporting the conclusions of this article will be made available by the authors, without undue reservation.
